# The mediating role of professional identity in the relationship between gender misconceptions and occupational stigma among male nursing students

**DOI:** 10.1186/s12912-025-03552-5

**Published:** 2025-07-17

**Authors:** Shaimaa Mohamed Amin, Doaa El Demerdash, Ahmed Abdellah Othman, Mohamed Ali Zoromba, Heba Emad El-Gazar, Mohamed Hussein Ramadan Atta, Ali Albzia, Mahitab Mohamed Abdelrahman, Ibrahim Alasqah, Haitham Mokhtar Mohamed Abdallah

**Affiliations:** 1 Lecturer of Community Health Nursing, Faculty of Nursing, Damanhour University, Damanhour City, Egypt; 2 Nursing Education Department, Faculty of Nursing, Damanhour University, Damanhour, Egypt; 3 Faculty of Nursing, Rashid University, Rashid, Egypt; 4 Lecturer of Nursing Administration Faculty of Nursing -Sohag University, Sohag, Egypt; 5 Psychiatric and Mental Health Nursing Department, Faculty of Nursing, Mansoura University, Mansoura, Egypt; 6 Nursing Administration Department, Faculty of Nursing, Port Said University, Port Said, Egypt; 7 Psychiatric and Mental Health Nursing, Alexandria University, Alexandria City, Egypt; 8 Nursing Department, College of Applied Medical Sciences, Prince Sattam bin Abdulaziz University, Wadi Aldawasir, Saudi Arabia; 9 Nursing Administration Department, Faculty of Nursing, Suez Canal University, Ismailia City, Egypt; 10 Department of Psychiatric and Mental Health, and Community Health, College of Nursing, Qassim University, Buraydah, 51452 Saudi Arabia; 11 Critical Care and Emergency Nursing, Faculty of Nursing, Alexandria University, Alexandria, Egypt; 12 Medical Surgical Department, College of Nursing, Jouf University, Sakaka, Saudi Arabia

**Keywords:** Male nursing, Students, Professional identity, Occupational stigma, Gender misconceptions, Nursing education, Egypt

## Abstract

**Background:**

Male nursing students often face unique challenges related to their professional identity, stigma associated with the nursing profession, and gender-related misconceptions. These factors can influence their academic and career development. Understanding these elements is crucial for improving the support systems and educational environments for male nursing students.

**Aim:**

This study aimed to assess the professional identity, occupational stigma, and gender misconceptions among male nursing students at Sohag University, Egypt, and explore the relationships between these factors.

**Methods:**

A cross-sectional descriptive design was used. A total of 308 male nursing students enrolled in the 2024–2025 academic year were selected through systematic random sampling. Data were collected using three validated instruments: the Professional Identity Scale for Male Nursing Students, the Nurse Occupational Stigma Scale, and The gender misconceptions of men in nursing scale. Descriptive statistics, Pearson’s correlation, and Linear regression analysis were employed to analyze the data. JASP 0.14.1.0 was used for testing the mediating role of male identity between der misconceptions and nurse occupational stigma through SEM Module through SEM Module with Delta method standard errors.

**Results:**

The participants’ mean score for professional identity was 38.5 (SD = 6.4). The mean score for the Nurse Occupational Stigma Scale was 53.2 (SD = 8.1), The GEMINI scale showed a mean score of 55.3 (SD = 7.9). In addition, revealed a significant positive correlation between professional identity and stigma (*r* = .52, *p* < .001), and a negative correlation between professional identity and gender misconceptions (*r* = -.45, *p* < .001). Also, stigma and gender misconceptions significantly predicted professional identity (R² = 0.38, *p* < .001).

**Conclusion:**

The study highlights significant associations between male nursing students’ professional identity, occupational stigma, and gender misconceptions. Addressing stigma and misconceptions can enhance the development of professional identity among male nursing students, fostering a more supportive academic environment.

**Clinical trial number:**

Not applicable.

## Introduction

The nursing profession occupies a complex intersection of gender norms, social identity, and occupational stigma, creating unique challenges for male nursing students who encounter biases deeply rooted in traditional gender expectations. Historically, nursing has been culturally linked to feminine characteristics such as nurturing, empathy, and caregiving, heavily influencing societal perceptions of the profession and fostering an implicit belief that men are inherently less suited for nursing [1, 2]. Consequently, male nursing students face entrenched stereotypes and biases that create barriers to their educational, professional, and personal growth. Central to this dynamic is the need to understand how gender misconceptions, occupational stigma, and professional identity interact, particularly how professional identity might mediate and mitigate the adverse effects of these biases. This study explores these complex relationships using scientific frameworks and proposes educational interventions to support male nursing students in building resilient professional identities [3, 4].

Gender misconceptions originate from societal beliefs about gender-specific roles, grounded in essentialist views that men and women possess distinct, biologically determined traits. Traditional gender role theory assigns men to assertive and independent roles, while women are deemed more suited to caregiving. Nursing, with its emphasis on empathy, compassion, and interpersonal connection, is thus perceived as a predominantly female profession [5, 6]. Men entering nursing challenge these norms and often experience occupational stigma negative social judgments directed at those who deviate from expected gender-normative career choices. For male nursing students, stigma manifests through microaggressions and doubts about their motives and competence. Research shows these experiences contribute to psychological distress, lower self-esteem, and increased stress. They also experience stereotype threat, wherein awareness of negative stereotypes causes anxiety and underperformance, reinforcing stigma. The psychological toll may lead to isolation, burnout, and attrition, highlighting the urgency of addressing occupational stigma to enhance resilience and success among male nursing students [7–9].

Professional identity, defined as an individual’s self-concept derived from roles, values, and responsibilities within a profession, plays a vital role in helping male nursing students manage and resist stigma. Social Identity Theory (SIT) and Role Identity Theory (RIT) offer frameworks to understand the protective function of professional identity [10]. Social Identity Theory (SIT) posits that self-worth derives from group affiliations such as professional identity, fostering resilience against societal bias and reinforcing belonging. For male nursing students, developing a strong professional identity affirms their legitimacy as healthcare providers, counteracting alienation from gender-based stigma [2, 10, 11]. RIT emphasizes that individuals’ role perceptions depend on social feedback. Male nursing students with a robust professional identity reinterpret caregiving as a skill-based, ethical practice consistent with nursing’s core values rather than conflicting with traditional masculine ideals. This realignment reduces stereotype threat, enhances psychological well-being, and promotes self-efficacy, supporting positive professional outlooks and persistence in nursing [11, 12].

Professional identity formation is a complex, ongoing process shaped by internal motivation and external influences. Clinical experiences, academic training, and interactions with peers and mentors significantly influence this process. Identity Theory suggests individuals construct self-concept by adopting roles that reflect their values, crucial for male nursing students navigating nontraditional career paths. Clinical exposure emphasizing skill mastery, patient interaction, and teamwork cultivates competence and belonging, offsetting negative stereotypes and stigma [12–14]. Mentorship, especially from nurses who have faced similar challenges, offers guidance to navigate gendered barriers and strengthens identity development. According to Social Learning Theory, observing and modeling mentor behavior helps male students incorporate nursing values into their self-concept. Research shows mentorship enhances resilience, professional pride, and belonging, boosting job satisfaction and retention [11, 12].

Redefining masculinity to include caregiving as a respected trait is another essential aspect of professional identity formation for male nursing students. Traditional masculinity is linked to strength, assertiveness, and independence, often seen as incompatible with nursing compassion and empathy. However, reframing masculinity within nursing to embrace a “heroic caregiver” model integrating protection, leadership, and empathy bridges traditional masculine traits with caregiving values. This reframing reduces cognitive dissonance, fosters identity congruence, and correlates with higher self-esteem and job satisfaction [8, 9]. Gender Role Conflict Theory explains that psychological strain emerges when personal identity conflicts with societal gender expectations [15]. By embracing caregiving as part of masculinity, male nursing students achieve identity congruence, reducing psychological strain and enhancing professional satisfaction. Studies find that male nurses who adopt this identity experience less occupational stigma, greater job satisfaction, and increased resilience to gender biases, leading to more positive Recognizing professional identity as a mediating factor in reducing occupational stigma has important implications for nursing education and healthcare policy. Educational institutions must foster inclusive environments that challenge gender stereotypes and actively support male nursing students’ identity development. Diversity and gender-sensitivity training for faculty, students, and staff can raise awareness of implicit biases and promote a culture that values male contributions, helping reduce stigma. Tailored mentorship programs addressing male students’ specific challenges support identity development, retention, and satisfaction [4, 16, 17]. Healthcare organizations and policymakers also play a pivotal role in promoting gender diversity. Increasing the visibility of male nurses through media campaigns, leadership initiatives, and public outreach challenges stereotypes and emphasizes nursing as a profession defined by skill and dedication rather than gender. Institutional support fosters environments where male nursing students feel valued and respected, decreasing occupational stigma and enhancing career satisfaction and longevity [16, 18].

In Egypt, the nursing profession has traditionally been dominated by female nurses. According to the latest data from the Central Agency for Public Mobilization and Statistics (CAPMAS, 2021), female nurses represent most of the workforce in both the public and private healthcare sectors. Specifically, in the public sector, females make up 91.1% of the workforce, with males comprising only 8.9%. In the private sector, the gender distribution is more balanced, with females representing 75.7% and males 24.3% [19, 20].

This study addresses the unique challenges male nursing students face due to gender misconceptions and occupational stigma, particularly the impact on professional identity formation and career satisfaction. Demographically, male nurses remain significantly underrepresented in the nursing profession, which has traditionally been dominated by females [19, 20]. Although the number of male nursing students has increased in recent years, they continue to encounter societal stigma and gender-based discrimination, often linked to their low representation in the field. Nursing’s traditionally female-dominated identity subjects’ male students to biases that disrupt their educational experience and sense of belonging [21].

Understanding how professional identity mediates these challenges is crucial to exploring the unique experiences of male nursing students in Egypt. Accordingly, this study aims to shed light on these issues, emphasizing the role of professional identity in navigating the intersection of gender misconceptions and occupational stigma. Professional identity acts as a crucial mediator, buffering stigma effects by fostering resilience, confidence, and commitment to nursing. Through identity development, male nursing students internalize nursing’s core values, helping them resist stigma and embrace caregiving as a respected, compatible role [5, 14].

Thus, this research investigates professional identity’s mediating role between gender misconceptions and occupational stigma among male nursing students. The findings contribute to advancing gender inclusivity in healthcare, providing policy and practice recommendations to improve retention and satisfaction among male nursing students. Ultimately, fostering diverse professional identities supports a more equitable, gender-neutral nursing workforce focused on skill and compassion rather than gendered stereotypes. Addressing the intersections of gender, stigma, and identity can help build a healthcare environment that empowers and includes all caregiver [9, 22, 23].

## Aim of the study

The aim of this study is to examine the mediating role of professional identity in the relationship between gender misconceptions and occupational stigma among male nursing students. Specifically, the study aims to explore how gender-related stereotypes and societal perceptions of nursing influence male students’ professional identity and how this, in turn, affects their experiences of occupational stigma within the nursing profession.

### Hypotheses

1. **H1**: There is a significant positive relationship between gender misconceptions and occupational stigma among male nursing students.
2. **H2**: Professional identity mediates the relationship between gender misconceptions and occupational stigma, such that stronger professional identity reduces the effect of gender misconceptions on occupational stigma.
3. **H3**: Male nursing students with a stronger professional identity will report lower levels of occupational stigma, even in the presence of gender misconception.

## Subjects and methods

### Study design and setting

This study utilized a cross-sectional descriptive research design, adhering to the Strengthening the Reporting of Observational Studies in Epidemiology (STROBE) guidelines. The research was conducted at the Faculty of Nursing, Sohag University, situated in Sohag Governorate, Egypt. The college functions under the supervision of the Egyptian Ministry of Higher Education and complies with national standards for nursing education. It consists of nine specialized departments and offers both undergraduate and graduate programs that operate on a credit hour system. This system provides a structured framework for monitoring academic progress and comprehensively assessing educational outcomes.

### Sample size and study participants

The target group for this study was male nursing students, selected based on specific inclusion criteria. Eligible participants were those currently enrolled in a nursing program during the academic year 2024–2025. To ensure adequate exposure to the nursing field, participants needed to have completed at least one semester of their nursing education. Additionally, only those who expressed a willingness to participate in the study were included. Participants were excluded if they were not currently enrolled as male nursing students or were enrolled in non-nursing healthcare programs. Those who had not completed at least one semester of nursing education were also excluded, as they might lack sufficient experience to contribute meaningfully to the study.

The sample size was established using the G*Power 3.1.9.7 software [23]. The calculation parameters were set at a power of 0.95, an alpha level of 0.001, and a moderate effect size of 0.15. These parameters indicated that a minimum of 304 participants was necessary. To accommodate potential dropouts, the sample size was increased to 320 participants.

**Fig. 1:**
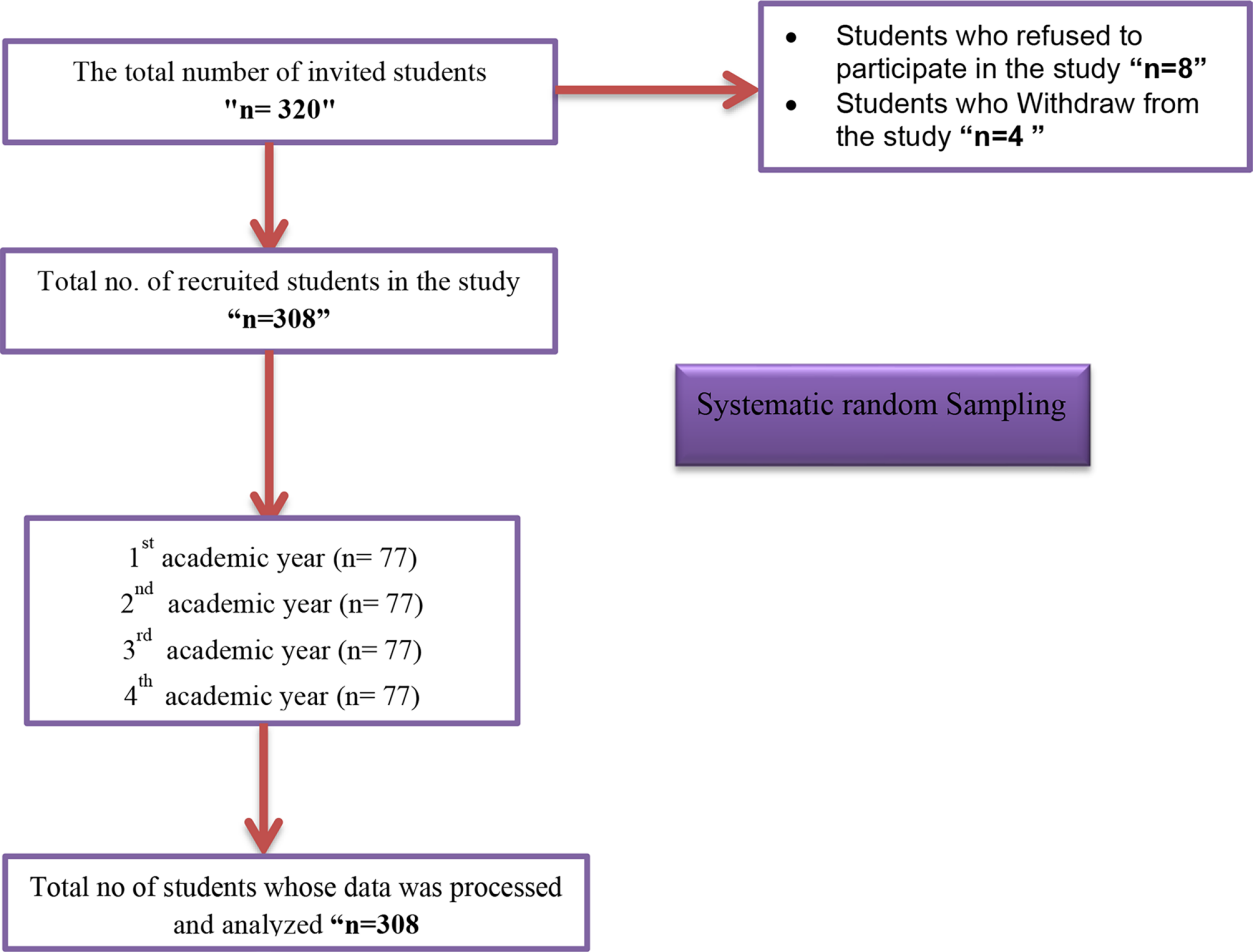
Flow chart of participants’ recruitment

The study employed an equal allocation sampling strategy to ensure balanced representation from each academic year, selecting 77 male nursing students from each of the four cohorts, totaling 308 participants. At the time of data collection, approximately 1,200 male nursing students were enrolled across all academic levels at the university. To achieve a representative sample, systematic random sampling was used by selecting every 30th student from an ordered list of eligible students, as illustrated in Figure 1. This method ensured randomness while maintaining proportionality and minimizing selection bias.

### Measurements of interest

#### The demographic form

The socio-demographic form, developed by the authors with reference to relevant literature, collected information including age, academic year, place of residence, family composition, income level, marital status, and parents’ educational background to ensure contextual relevance and comprehensive participant profiling [18–20].

#### Professional identity scale for male nursing students

It was developed by Li and Lou in 2022 to assess the professional identity of male nursing students [24]. The scale consists of 12 items distributed across three dimensions: Cognitive Identity (4 items), Emotional Identity (4 items), and Behavioral Identity (4 items). Each item is rated on a five-point Likert scale, ranging from strongly disagree [1] to strongly agree [5], with higher total scores indicating a stronger professional identity. The scale demonstrated a good model fit in confirmatory factor analysis, with factor loadings ranging from 0.56 to 0.73. Cronbach’s alpha coefficients ranged from 0.72 to 0.79 for the three subscales and 0.88 for the entire scale. In this study, the scale showed high reliability, with a Cronbach’s alpha of 0.91. After translation into Arabic, exploratory factor analysis (EFA) confirmed its validity, with factor loadings ranging from 0.58 to 0.89, improving to 0.70 to 0.94 post-varimax rotation, and accounting for 78.350% of the variance. The Kaiser–Meyer–Olkin (KMO) measure was excellent at 0.950, and Bartlett’s test of sphericity was highly significant (*p* ≤ .001), confirming the data’s suitability for factor analysis. All items on the scale were retained.

#### The nurse occupational stigma scale (NOSS)

The Nurse Occupational Stigma Scale (NOSS) was developed by Yang et al. (2022) to assess the level of stigma associated with the nursing profession. The scale consists of 16 items divided across three dimensions: Negative Label (5 items), Nurse-Patient Relationship (6 items), and Devaluation and Discrimination (5 items). The Negative Label dimension includes items such as “Nurses are less educated” and “Nurses act as a servant to people,” capturing stereotypes that frame nurses as lacking professional competence or status. Participants respond using a 5-point Likert scale ranging from 1 (strongly disagree) to 5 (strongly agree), with higher scores indicating stronger perceptions of occupational stigma. In terms of psychometric properties, the original scale showed excellent internal consistency, with Cronbach’s alpha of 0.920. Confirmatory Factor Analysis (CFA) supported the three-factor structure, with acceptable fit indices (χ²/df = 2.635, RMSEA = 0.064, and CFI = 0.962). After translation into Arabic, the scale maintained strong reliability (Cronbach’s alpha = 0.910). Exploratory Factor Analysis (EFA) further supported construct validity, with factor loadings ranging from 0.50 to 0.90, which improved to 0.65 to 0.98 after varimax rotation, accounting for 74.216% of the variance. The Kaiser–Meyer–Olkin (KMO) measure was 0.940, and Bartlett’s test of sphericity was highly significant (*p* ≤ .001), confirming the adequacy of the data for factor analysis. All items were retained in the final version of the scale.

#### The gender misconceptions of men in nursing (GEMINI) scale

The Scale was developed by Montayre et al. (2022) to assesses nursing students’ perceptions of gender-related stereotypes about male nurses [26]. It comprises 17 items rated on a five-point Likert scale from strongly disagree [1] to strongly agree [5], with higher scores indicating greater gender misconceptions about men in nursing. According to Montayre et al. (2022), the scale demonstrated strong content validity, with an item content validity index (I-CVI) exceeding 0.70 for retained items. Exploratory Factor Analysis (EFA) initially revealed factor loadings between 0.31 and 0.66, which improved to 0.60 to 0.90 after varimax rotation, with a one-factor solution accounting for approximately 70% of the variance. Confirmatory Factor Analysis (CFA) supported the scale’s validity with χ² = 220.24, df = 92, CFI = 0.967, and RMSEA = 0.047. The scale showed high internal consistency in our study, with Cronbach’s alpha of 0.90. The Kaiser-Meyer-Olkin (KMO) measure was robust at 0.90, and Bartlett’s test of sphericity was highly significant (*p* ≤ .001), confirming the data’s suitability for factor analysis.

## Study procedures

### Tool Preparation & pilot study

The research instruments, including Professional Identity Scale for Male Nursing Students, NOSS and GEMINI were translated into Arabic by bilingual experts with proficiency in both English and Arabic. We emphasized accuracy and cultural relevance throughout the translation process. To validate these translations, they were rigorously back-translated into English to ensure linguistic equivalence and identify any discrepancies. Following this, face validity assessments were conducted for each instrument, with expert panels meticulously reviewing the translated tools to confirm they captured the intended constructs accurately within the Arabic context. Additionally, feedback was gathered from potential participants to evaluate the clarity, relevance, and cultural appropriateness of the translated items. Reliability was assessed using statistical methods, such as Cronbach’s alpha, to ensure internal consistency. A pilot study involving 30 participants was then conducted to test the translated instruments’ clarity, relevance, and reliability. These participants were subsequently excluded from the main study. The results of the pilot study indicated that no further modifications to the instruments were necessary.

### Data collection

Data collection for this study took place between September and October 2024, following the necessary approvals. Before starting, the researchers provided each student with an explanation of the study’s objectives, highlighting that participation was voluntary. Written informed consent was obtained from all participants before they took part in the study. To build trust, the researchers assured participants that their responses would remain confidential. The questionnaires were distributed in quiet environments, such as empty lecture halls and libraries, between 9 a.m. and 2 p.m., from Saturday to Thursday. On average, participants took 10 to 15 min to complete each questionnaire.

### Ethical considerations

Approval was obtained from the Research Ethics Committee of the Faculty of Nursing at Sohag University, Egypt, with reference number (191). The study adhered to ethical principles outlined in the Declaration of Helsinki, ensuring the protection of participants’ rights and well-being. All participants provided written informed consent following a thorough explanation of the study’s objectives. Participants’ privacy and anonymity were rigorously protected, and all data collected was treated with the utmost confidentiality. Furthermore, participants were informed that they had the right to withdraw from the study at any time.

### Data analysis

SPSS 26.0 (IBM Inc., Chicago, IL, USA) was used for data analysis was performed to evaluate the survey responses from the 308 nursing students. The participants’ general characteristics and the scores obtained on various scales descriptive statistics, were evaluated frequencies (No/%) and mean ± standard deviations (S.D.), were utilized to summarize. The correlations between male identity, der misconceptions and nurse occupational stigma were examined by Pearson’s correlation analysis utilized to assess. Linear Regression analysis test used to assess relation between variables and personal characteristics among participants JASP 0.14.1.0 was used for testing the mediating role of male identity between der misconceptions and nurse occupational stigma through SEM Module with Delta method standard errors.

## Results

**Table 1:** Personal characteristics among participants (*n* = 308)

Personal data	Categories	No	%
Age	less than 20 years	204	66.2
	20 to less than 22	90	29.2
	22 to less than 24	11	3.6
	more than 24	3	1.0
Residence	Rural	146	47.4
	Urban	162	52.6
Monthly Income	Not enough	44	14.3
	Enough	203	65.9
	enough and save	61	19.8
Marital status	single	303	98.4
	married	5	1.6
Mother education	illiterate	45	14.6
	basic education	73	23.7
	secondary	102	33.1
	university and more	88	28.6
Father education	illiterate	30	9.7
	basic education	79	25.6
	secondary	86	27.9
	university and more	113	36.7

Table 1: Shows that 66.2% of the participants had less than 20 years, 52.6% of them are from urban areas and 65.9% of them had enough monthly income. Also, 98.4% of them were single, 33.1% of them their mother had secondary education and 36.7% of them their father had university and more education.

**Table 2:** Descriptive analysis between study variables (*n* = 308)

Variables	*N*	Minimum	Maximum	Mean ± SD	Average Mean ± SD
Cognitive Identity	4	6.00	19.00	15.8831 ± 1.84743	3.9708 ± 0.46186
Emotional Identity	4	5.00	20.00	17.5779 ± 2.81754	4.3945 ± 0.70439
Behavioral Identity	4	5.00	20.00	15.2208 ± 1.97458	3.8052 ± 0.49365
**Male identity**	12	16.00	56.00	48.6818 ± 5.59456	4.0568 ± 0.46621
Negative Labe	5	5.00	25.00	11.2532 ± 5.35522	2.2506 ± 1.07104
Nurse-Patient Relationship	6	6.00	28.00	13.6753 ± 5.48719	2.2792 ± 0.91453
Devaluation and Discrimination	5	6.00	25.00	13.7013 ± 4.64664	2.7403 ± 0.92933
**Nurse Occupational Stigma**	16	17.00	76.00	38.6299 ± 14.34814	2.4144 ± 0.89676
**Gender misconceptions**	17	17.00	85.00	44.6299 ± 17.13273	2.6253 ± 1.00781

SD = Standard Deviation

Table 2: Reveals that, the average mean score of the participants male identity scale was 4.0568 ± 0.46621 with the subscales were 3.9708 ± 0.46186 for cognitive identity, 4.3945 ± 0.70439 for emotional identity, 3.8052 ± 0.49365 for behavioral identity. Furthermore, the average mean score of the participants nurses occupational stigma scale was 2.4144 ± 0.89676 with the subscales; negative Labe was 2.2506 ± 1.07104, nurse-patient relationship was 2.2792 ± 0.91453 and 2.7403 ± 0.92933 for devaluation and discrimination. In addition, the average mean score of the participants’ gender misconceptions was 2.6253 ± 1.00781.

**Table 3:** Linear regression analysis between variables (*n* = 308)

Regression equation		Overall fitness index			Significance of regression coefficient			
Outcome variables	Predictive variable	*R*	*R* ^2^	F	B	Beta	t	Sig.
Male identity	Age	0.346	0.120	6.834	0.461	0.050	0.907	0.365
	Residence				− 0.953	− 0.085	-1.545	0.123
	Monthly Income				-1.576	− 0.164	-3.022	**0.003**
	Marital status				0.268	0.006	0.110	0.912
	Mother education				-1.661	− 0.304	-4.484	**0.000**
	Father education				0.342	0.061	0.910	0.364
Nurse Occupational Stigma		0.565	0.306	23.557				
	Age				0.548	0.023	0.479	0.632
	Residence				-1.009	− 0.035	− 0.725	0.469
	Monthly Income				1.027	0.042	0.873	0.383
	Marital status				-4.304	− 0.038	− 0.783	0.434
	Mother education				0.275	0.020	0.329	0.742
	Father education				-8.199	− 0.574	-9.683	**0.000**
Gender misconceptions		0.490	0.241	15.890				
	Age				− 0.761	− 0.027	− 0.527	0.599
	Residence				− 0.015	0.000	− 0.008	0.993
	Monthly Income				3.448	0.117	2.324	**0.021**
	Marital status				4.971	0.037	0.717	0.474
	Mother education				3.234	0.194	3.069	**0.002**
	Father education				-9.609-	− 0.563-	-8.996-	**0.000**

Table 3: Shows that the regression model for predicting factors for male identity was (*R* = .346, R^2^ = 0.120 & F = 6.834) with statistical significant difference between male identity and monthly income at (B=-1.576, Beta=-0.164, t=-3.022, *P* = .003) and mother education at (B=-1.661, Beta=-0.304, t=-4.484, *P* = .000). Regarding the Nurse Occupational Stigma predicting model there was (*R* = .565, R^2^ = 0.306 & F = 23.557) with statistically significant difference between Nurse Occupational Stigma nd father education at (B=-8.199, Beta=-0.574, t=-9.683, *P* = .000). Regarding Gender misconceptions predicting model there was (*R* = .490, R^2^ = 0.241 & F = 15.890) with statistical significant difference between Gender misconceptions and monthly income at (B = 3.448, Beta = 0.117, t = 2.324, *P* = .021) and mother education at (B = 3.234, Beta = 0.194, t = 3.069, *P* = .002) and father education at (B=-9.609, Beta=-0.563, t=-8.996, *P* = .000).

**Table 4:** Correlation analysis between study variables (*n* = 308)

Variables		1	2	3	4	5	6	7	8	9
Cognitive Identity (1)	r	1								
Emotional Identity (2)	r	0.519^**^	1							
Behavioral Identity (3)	r	0.471^**^	0.649^**^	1						
**Male identity (4)**	r	0.757^**^	0.904^**^	0.835^**^	1					
Negative Labe (5)	r	− 0.133^*^	− 0.419^**^	− 0.130^*^	− 0.301^**^	1				
Nurse-Patient Relationship(6)	r	− 0.102	− 0.324^**^	− 0.088	− 0.228^**^	0.846^**^	1			
Devaluation and Discrimination(7)	r	− 0.167^**^	− 0.273^**^	− 0.059	− 0.213^**^	0.763^**^	0.740^**^	1		
**Nurse Occupational Stigma(8)**	r	− 0.143^*^	− 0.369^**^	− 0.101	**− 0.269** ^**^	0.944^**^	0.938^**^	0.892^**^	1	
**Gender misconceptions(9)**	r	− 0.084	− 0.383^**^	− 0.192^**^	**− 0.288-** ^**^	0.282^**^	0.255^**^	0.051	**0.220** ^**^	1

**Correlation is highly significant at the 0.01 level (2-tailed)

Table 4: Shows that there was high statistical significant negative correlation between male identity and nurse occupational stigma (*r* = -.269^**^) and between male identity and gender misconceptions at (*r* = −.288^**^), while there was positive correlation between nurse occupational stigma and gender misconceptions at (*r* = .220^**^).

**Table 5:** Mediating effect of male identity between gender misconceptions and nurse occupational stigma (*n* = 308)

Direct effect	(B)	CI 95%	t	*p*
Gender misconceptions → male identity	− 0.094	(-0.129–0.059)	5.266	< 0.001^**^
Gender misconceptions → Nurse Occupational Stigma	0.130	(0.036-0.224)	2.723	0.007^**^
male identity → Nurse Occupational Stigma	− 0.575	(-0.862-0.287-)	-3.937-	< 0.001^**^
**Indirect effect**				
Gender misconceptions → male identity → Nurse Occupational Stigma	0.054	(0.021–0.088)	3.167	0.002 ^**^
**Total effect**				
Gender misconceptions → Nurse Occupational Stigma	0.184	(0.093–0.275)	3.950	< 0.001^**^

**Fig. 2:**
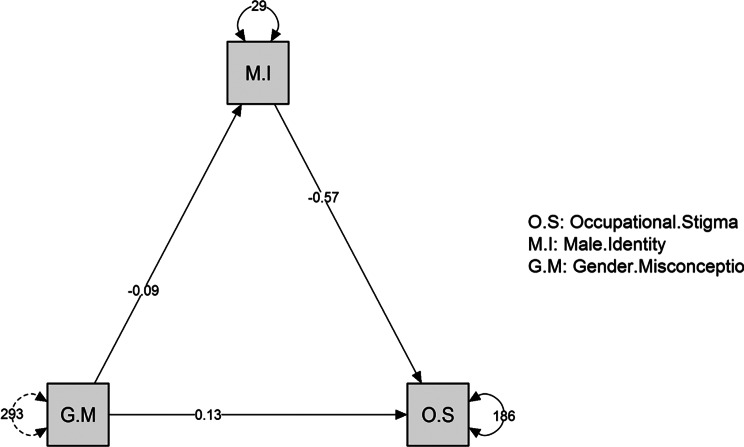
Mediating effect of male identity between Gender misconceptions and Nurse Occupational Stigma (*n* = 308)

4.5. Mediation Analysis. Table 5; Figure 2: Illustrates that, there was a statistically significant direct effect of gender misconceptions on male identity at (B=-0.094, t = 5.266, < 0.001), on nurse occupational stigma at (B = 0.130, t = 2.723, 0.007^**^) and male identity on nurse occupational stigma at (B=-0.575, t=-3.937-, < 0.001). Furthermore, there was a statistically significant indirect effect of gender misconceptions on nurse occupational stigma when the male identity act as a mediator variable at (B = 0.054, t = 3.950, < 0.001).

## Discussion

This study aimed to investigate the role of professional identity as a mediating factor in the relationship between gender misconceptions and occupational stigma experienced by male nursing students. The results demonstrated a positive relationship between gender misconceptions and nurse occupational stigma among male nursing students. This crucial finding highlights that addressing gender misconceptions is associated with a decrease in occupational stigma among male nursing students. The persistence of traditional gender stereotypes associating nursing with femininity leads to misconceptions about male nurses’ suitability for the profession, thereby creating a perceived incongruity of men in the nursing profession, reducing their confidence in pursuing their roles within the nursing field, and increasing their vulnerability to stigma [24]. These results align with prior studies that have shown how challenging gender stereotypes can positively impact reduce stigma in traditionally gendered professions [25]. Additionally, these findings are consistent with a nursing study by Hung et al. (2019), which suggests that gender-role barriers contribute to a diminished nursing image among male students [26].

Concerning the relationship between gender misconceptions and professional identity among male nursing students, we found that gender misconceptions are significantly associated with a diminished professional identity. Over recent decades, various factors, such as self-esteem, psychological distress, and heavy workload, have been identified as barriers to male nursing students’ professional identity formation [12, 27]. We have introduced a novel perspective highlighting how ingrained gender misconceptions directly impact the professional identity of male nursing students. Our findings align with previous research indicating that gender stereotypes can negatively influence male personal self-esteem [28]. Similarly, Minehart et al. (2020) found that addressing misconceptions and increasing awareness of gender inclusivity could enhance nurses’ confidence and collaboration [29].

The findings also demonstrated that male nursing students’ professional identity is negatively related to their sense of occupational stigma. This indicates that when male nursing students have a strong and well-developed professional identity, they are likely to experience lower levels of occupational stigma. A robust professional identity helps male nursing students feel confident and valued in their roles, mitigating the impact of societal stereotypes and misconceptions [30, 31]. This confidence can reduce their perception of stigma and enable them to embrace their roles in the nursing profession more fully [32]. These results align with those of Doldor and Atewologun (2020) who found that fostering a positive self-concept can reduce stigma [33]. Similarly, [34] emphasized that a strong professional identity empowers male nursing students to redefine their roles within the nursing field [34].

The study revealed a significant effect of paternal education on male nursing students’ occupational stigma. Higher paternal education levels were associated with reduced stigma, likely because educated fathers in Egypt often exposed to professional networks—recognize nursing as a respected career. They perceive nursing as part of a global profession where nurses attend conferences, publish research, and pursue higher education. Additionally, fathers prioritize financial stability, viewing nursing as a secure career in Egypt’s competitive job market. This contrasts with traditional views that undervalue the profession. These findings align with Dos Santos (2020) [35], who found that parental education influences nursing students’ career choices. However, they contradict a study in China, which showed no significant link between paternal education and career perception [27], possibly due to cultural and socioeconomic differences.

The study also found significant effects of paternal and maternal education on male nursing students’ gender misconceptions. Higher paternal education reduced misconceptions, likely because educated fathers in Egypt are more open to gender equality, encouraging their sons to pursue nursing. In contrast, higher maternal education increased gender misconceptions, possibly because educated mothers perceive nursing’s demanding work environments such as night shifts or critical care units as unsuitable for men, reinforcing the stereotype that nursing is a female-dominated profession. This aligns with Raghavan et al. (2023) who noted that parental education shapes gender perceptions in nursing [36]. Additionally, the findings relate to research on barriers faced by Jordanian nursing students [37], further highlighting the influence of parental attitudes on gender misconceptions. Awareness campaigns could help parents better support male nursing students.

Lastly, the findings of this study suggest that male professional identity serves as a partial mediator in the relationship between gender misconceptions and nurse occupational stigma, accounting for approximately 29.3% of the effect (indirect effect B = 0.054, 95% CI [0.021, 0.088], *p* = .002). This implies that when male nursing students develop a strong sense of professional identity, despite facing gender misconceptions, they are better equipped to overcome the stigma associated with their roles. A well-developed professional identity enables male nursing students to challenge stereotypes, affirm their value within the nursing profession, and diminish the impact of occupational stigma. These findings align with prior research in related fields, which indicates that fostering a positive self-concept and professional identity can mitigate the negative effects of societal biases and enhance resilience against stigma [12]. Additionally, this mediation model is consistent with previous nursing research, which found that professional identity acts as a mediating mechanism between difficulties stemming from the nursing profession and nurses’ stress [38]. Furthermore, while this model is novel in the context of male nursing students, it parallels findings from other professions. For instance, research on teachers’ professional identity has shown that a strong professional self-concept helps counteract societal devaluation and occupational stigma [39].

### Strengths and limitations

This study design, utilizing a cross-sectional descriptive approach, offers several strengths, particularly its ability to gather a broad, representative sample of male nursing students from different academic years. The systematic random sampling method ensures that the sample is unbiased, with 320 participants providing a solid basis for analysis and enhancing the study’s generalizability. The inclusion of well-established and validated scales, such as the Professional Identity Scale for Male Nursing Students, the Nurse Occupational Stigma Scale (NOSS), and the GEMINI scale, ensures that the data collected accurately reflects the constructs of professional identity, stigma, and gender misconceptions. Furthermore, the use of a rigorous translation and back-translation process, along with pilot testing, strengthens the reliability and cultural appropriateness of the instruments, enhancing the quality of the data.

However, studying also has notable limitations. Cross-sectional design, while efficient in collecting data at a single point in time, limits the ability to establish causal relationships between variables. The study’s focus on male nursing students at a single university in Egypt means that the findings may not be fully generalizable to male nursing students in different regions or countries. Additionally, the exclusion of non-nursing healthcare students introduces potential selection bias, as the experiences of male students in other healthcare fields may differ. These limitations suggest that while the study provides valuable insights, further research using longitudinal designs and more diverse samples would be beneficial to explore the factors influencing male nursing students’ professional identity, stigma, and gender misconceptions over time and across different settings.

### Recommendations

The study highlights the significant impact of gender misconceptions and parental education on male nursing students’ identity and the occupational stigma they experience. To address these challenges, nursing education programs should incorporate targeted interventions that challenge gender stereotypes and promote positive male professional identity. Mentorship and peer support initiatives specifically designed for male nursing students can strengthen their sense of belonging and resilience against stigma. Additionally, family and community awareness campaigns are crucial, especially focusing on educating parents to foster acceptance of nursing as a respectable career path for men. Financial support mechanisms should also be considered, as socioeconomic factors play a role in shaping male identity and perceptions.

Moreover, efforts to improve public and media representation of male nurses are essential to counteract negative labels and misconceptions. Collaborations with media outlets to showcase diverse and successful male nursing professionals can help normalize men in caregiving roles and reduce societal stigma. Nursing institutions should implement inclusive policies that promote gender diversity, ensure equal opportunities, and provide a supportive environment free from discrimination. Continued research and monitoring are needed to evaluate the effectiveness of these strategies and to tailor culturally sensitive interventions that further dismantle occupational stigma among male nurses.

### Implications

The findings of this study open several avenues for future research in nursing, particularly in understanding the intersection of gender misconceptions and professional identity. Researchers can explore how these factors influence the mental health and career satisfaction of male nursing students and professionals. Future studies may also investigate the role of gender misconceptions in other healthcare professions, examining whether similar patterns of stigma exist in different settings. Additionally, longitudinal studies could assess how male nursing students’ experiences with occupational stigma evolve throughout their careers and the long-term impact on their professional identity. Investigating interventions aimed at reducing gender misconceptions and promoting positive male identities in nursing could further enrich the field.

Nursing education programs should prioritize the development of a positive professional identity among male nursing students. Educators can implement curricula that challenge traditional gender stereotypes and emphasize the value of diversity in the profession. Training programs could include discussions on gender misconceptions, stigmatization, and strategies for fostering inclusivity. Mentorship programs led by male nursing role models can also help male students navigate the challenges associated with occupational stigma, promoting confidence and resilience. Educators must be equipped to recognize the subtle effects of gender bias and be proactive in creating an environment that supports the professional growth of male nursing students.

In clinical practice, addressing occupational stigma and promoting a positive professional identity is crucial for enhancing job satisfaction and retention rates among male nurses. Healthcare organizations should foster an inclusive environment that actively works to combat gender-based biases and misconceptions, encouraging all nurses, regardless of gender, to feel valued and supported. Furthermore, nursing leaders should advocate for policies that promote gender equality, ensuring that all nurses, particularly males, have equal opportunities for career advancement. Training staff to recognize and challenge occupational stigma can contribute to a more supportive work environment, ultimately benefiting patient care by ensuring a diverse and well-supported nursing workforce.

## Conclusion

The findings of this study demonstrate a significant relationship between gender misconceptions and occupational stigma among male nursing students. Additionally, gender misconceptions are negatively associated with professional identity, while male nursing students’ professional identity is inversely related to their experience of occupational stigma. This study reveals that professional identity serves as a critical mediator between gender misconceptions and occupational stigma, supporting and extending Social Identity Theory within nursing contexts.

## Data Availability

The datasets generated and analyzed during the current study are not publicly available due to confidentiality agreements but are available upon reasonable request from the corresponding author.
